# Probiotics and palmitoylethanolamide (PEA) for osteoarthritic pain: individual effects in a multiple baseline design study

**DOI:** 10.1186/s12906-025-05187-0

**Published:** 2026-02-19

**Authors:** Isabelle Taye, Joanne Bradbury, Sandra Grace

**Affiliations:** Southern Cross University, Lismore, Australia

**Keywords:** Osteoarthritis, Palmitoylethanolamide, Probiotics, Complementary medicine, Alternative medicine, Multiple baseline design trial

## Abstract

**Background/Objectives:**

Osteoarthritis (OA) is a leading cause of chronic pain. As healthcare services struggle to meet demand to treat chronic pain, many individuals self-manage their symptoms. Probiotics and palmitoylethanolamide (PEA) may be promising therapeutic options because of their anti-inflammatory properties. PEA is an endogenously produced N-acylethanolamine, considered “endocannabinoid-like” due to its structural similarity to endocannabinoids. While it has non-direct influence on the endocannabinoid system, it primarily acts via non-cannabinoid pathways, most notably through activation of peroxisome proliferator-activated receptor alpha (PPARα). PEA has demonstrated both analgesic (pain-relieving) and mood-modulating effects in preclinical studies, and preliminary clinical studies. To date, no clinical studies have investigated the combined use of PEA and probiotics for the treatment of OA pain.

**Methods:**

A multiple baseline design (MBD) study was used over 11 weeks to assess the individual effects in four participants recruited from a naturopathic practice. Participants were randomised into one of two pathways, both starting with a placebo phase, followed by an active intervention involving probiotics and PEA. This design allowed for the concealment and blinding of the introduction of active treatment in a double-blind manner. The primary outcome was daily pain scores using a Visual Analogue Scale (VAS). Secondary outcomes incorporated a patient-reported measure which was a patient specified functional scale. Other secondary outcomes assessed wellbeing, stress, and blood indicators of inflammation. Visual analysis of time series graphs and descriptive statistics were used to analyse the data.

**Results:**

The graph demonstrated a clear pain reduction for one participant. Analyses also suggested improvements in patient-specified functional scales, wellbeing, and anxiety for all participants.

**Conclusions:**

This small multiple‑baseline study suggests that a probiotics‑plus‑PEA regimen may support function and wellbeing in some individuals with OA; these preliminary, hypothesis‑generating findings warrant evaluation in larger, controlled studies.

**Trial registration:**

This trial was registered with the Australian New Zealand Clinical Trials Registry on the 18th of January 2021. The registration number is ACTRN#:12621000039886.

## Introduction

### Osteoarthritis and individuals

Osteoarthritis (OA) is a common musculoskeletal disorder characterized by cartilage remodelling and inflammation, leading to pain and disability [1, 2]. As a leading cause of chronic pain, it is also associated with changes in behaviour, sleep, and mood [3]. Risk factors include age, female gender, obesity, and repeated stress on joints from overuse [4, 5]. A recent national survey of individuals with chronic pain reported most respondents did not feel they received adequate care from available health services [6]. Many current medications focus on managing symptoms of pain and inflammation, though some may also target underlying processes. However, these treatments often have adverse side effects [1, 5]. As pain is an individualised experience, greater research that investigates individual impacts of novel treatment options on pain is warranted. Recent research suggests that the gut microbiome, which is unique to individuals, may play a role in inflammation, potentially influencing OA development and progression [7].

### Probiotics

Recent advances in knowledge into the microbiome suggest it may be involved in disease genesis through immune system interaction [8, 9]. According to a review in 2022 [7], the microbiome may influence ageing, obesity, and inflammation. It is postulated that pathological bacteria may alter immune function resulting in chronic, low grade, systemic inflammation [8]. For example, anaerobic bacteria such as *Bilophila* have been associated with inflammation and OA [7]. Therefore, through the “gut-joint axis” [10–12], probiotics may reduce pain and inflammation, improve gastrointestinal integrity, and increase bone metabolism [13–15].

Numerous studies in recent years have focused on probiotics and their ability to attenuate a variety of health conditions including metabolic and gastrointestinal disease [16]. As probiotics are available through food sources and supplementation and have been used with concomitant medication for other conditions, they are generally regarded as safe with adverse effects being rare [16, 17]. Commonly available strains of probiotics include *Lactobacillus*,* Bifidobacterium*, and *Saccharomyces cerevisiae* [16]. *Lactobacillus* strains *plantarum*,* rhamnosus*,* reuteri*, *agilis*, and *Bifidobacterium animalis* ssp. *lactis* [18] are thought to reduce inflammation through cellular and humoral immunity [19]. Consequently, probiotics may provide a novel treatment option to slow disease progression and attenuate symptoms in individuals experiencing chronic pain from OA [20].

### Palmitoylethanolamide (PEA)

Palmitoylethanolamide (PEA) is an endogenously produced N-acylethanolamine and is considered ‘endocannabinoid-like’ due to its structural similarity to endocannabinoids such as anandamide, and its ability to modulate the endocannabinoid system through indirect mechanisms [21]. PEA is not a classical endocannabinoid per se as it does not directly bind CB1 or CB2 receptors. Instead, its principal target is proliferator-activated receptor-alpha (PPARα), with additional interactions involving TRPV1 and GPR55, through which it modulates inflammatory and nociceptive signalling and may influence mood [6, 22]. PEA may exert anti-inflammatory effects through downregulating inflammatory gene expression and reducing the production of inflammatory cytokines that are involved in cartilage destruction [21]. Further, PEA may be able to stabilize local mast cells to prevent the release of inflammatory mediators such as histamine and TNF-α that drive synovitis and pain [23]. PEA’s analgesic properties may be the result of its ability to interact with multiple targets, including the TRPV1 channel, to desensitize pain-sensing nerve fibers [23].

Regardless of whether PEA is produced endogenously or synthetically, it appears to offer similar effectiveness in treating chronic neuropathic pain, inflammation, stress, and anxiety [24, 25]. Endogenously, PEA production is tightly regulated [26] and made on-demand to act locally [27]. PEA can also be found in food sources such as lecithin and soybeans [28]. As a synthetic compound, PEA can be supplemented orally with daily dosages in clinical trials ranging from 300 to 1200 mg for chronic pain [24, 29]. To date there have been no reported issues of PEA dependence, withdrawal or addiction [30]. Furthermore, PEA seems well tolerated with no known contraindications with medications or supplements [29, 31].Therefore, PEA’s multimodal actions (PPARα activation with additional interactions at TRPV1 and GPR55) support anti‑inflammatory and analgesic effects relevant to low‑grade synovitis, mast‑cell activation, and nociceptive sensitization noted in OA [32]. Interestingly, it may also influence mood via these pathways. Taken together, these features make PEA a plausible and novel adjunct to conventional OA management, particularly where pain and low mood co‑occur. In this study, we primarily sought anti‑inflammatory and analgesic effects relevant to OA pathology, with mood outcomes prespecified as exploratory secondary measures.

### Study rationale and aim

To address the need for more personalised OA management, together with the lack of clinical studies evaluating combined probiotics and PEA, the aim was to conduct a double‑blind, multiple‑baseline study with a placebo lead‑in to assess their combined effects on pain, function, and wellbeing in individuals with chronic osteoarthritis pain. In this study, we primarily sought anti-inflammatory and analgesic effects of PEA relevant to OA pathology, with mood outcomes assessed as exploratory secondary measures. This study builds on a previous N-of-1 trial that demonstrated probiotics were associated with a reduction in OA pain and improvements in selected aspects of functioning for a single individnullual [20]. By using a MBD study, participant burden is lessened by eliminating the need for multiple, lengthy withdrawal periods (as required in N-of-1 studies). Additionally, the inclusion of multiple participants can help identify trends within each individual and also across the cohort.

## Materials and methods

### Participants

**Fig. 1:**
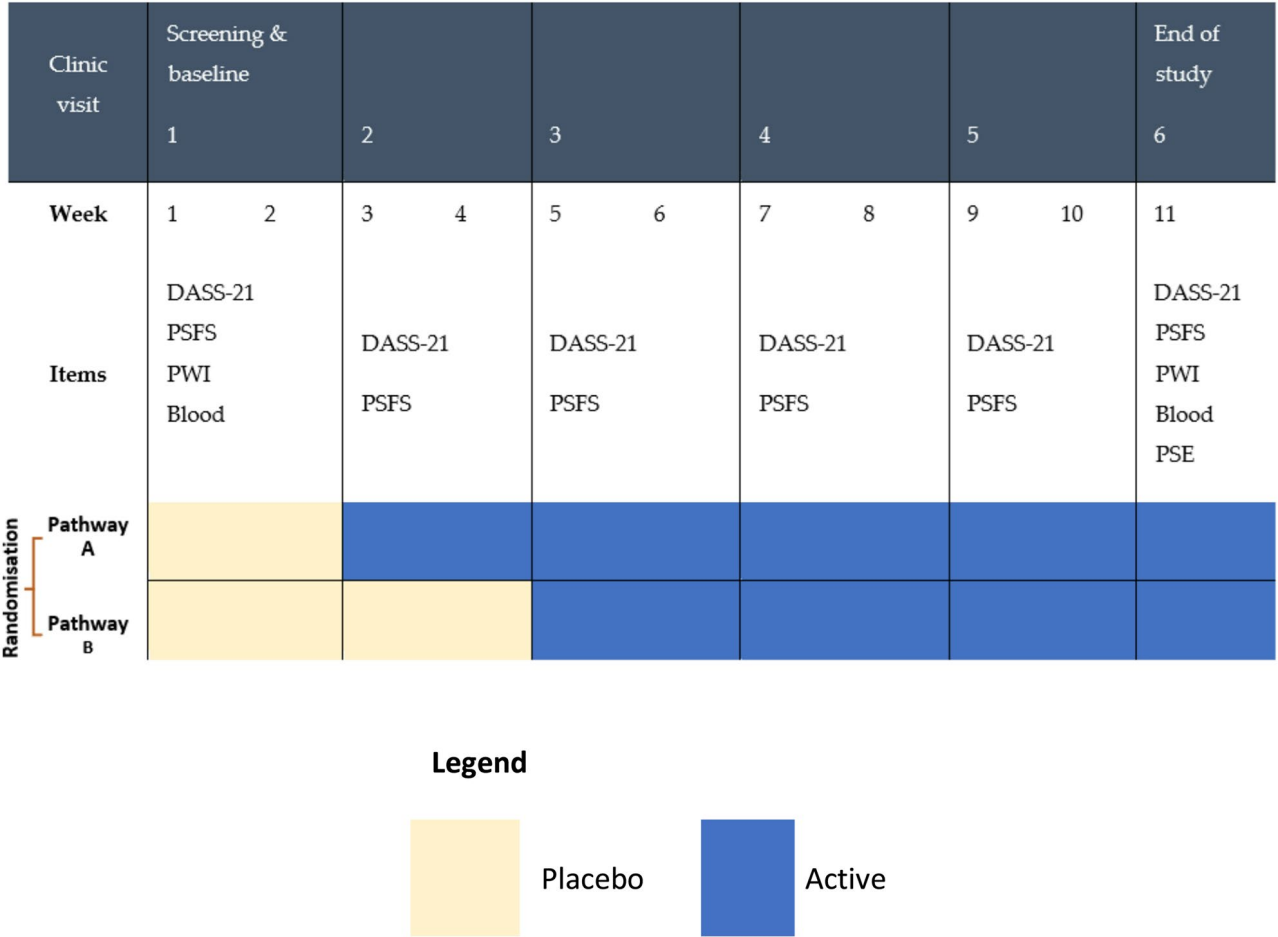
Multiple baseline design showing two pathways. Note: pathway a had a baseline phase of 2 weeks; pathway B had a baseline phase of 4 weeks. Abbreviations: DASS-21 - Depression Anxiety Stress Scale, PSFS – Patient Specific Functional Scale, PWI – Personal Wellbeing Index, Blood - blood test including full blood count, vitamin D, iron, C-Reactive Protein, erythrocyte sedimentation rate and fibrinogen, PSE – participant study experience

Participants were recruited from a private naturopathic clinic in Sydney, Australia. Participants were selected if they met the following criteria: aged between 18 and 85, a medical diagnosis of OA and self-identifying as experiencing psychological stress. Exclusion criteria were: history of physical trauma to the OA-affected joints, presence of other inflammatory joint conditions, chronic gastrointestinal conditions, diabetes, compromised immune system, and females who were lactating, pregnant or planning to become pregnant. Individuals were also excluded if they were taking blood thinning medications, antidepressant medication for less than 6 months, or had experienced psychological side effects due to antidepressant medications. As the MBD involved two pathways (Fig. 1), the study was designed to trial the individual effects of two participants per pathway, with a total of four participants being sought for the study.

### Research design and protocol

An MBD study was conducted over 11 weeks. The time to treatment was randomised across two pathways (Fig. 1). Both pathways began with a placebo phase, followed by an active intervention phase. Both phases involved oral administration of supplements, either probiotics and PEA or matched placebos. This design enabled double-blinding of the treatment phase, as both participants and the outcomes assessor were blinded to the study design. Baseline phases of two or four weeks were used to establish individual trajectories prior to randomised introduction of the active phase; baseline stability was monitored pragmatically rather than pre-specified as a formal criterion for progression. This study was registered with the Australian New Zealand Clinical Trials Registry ACTRN#:12621000039886.

Two participants began active interventions at the second clinic visit (Week 3 in Fig. 1) while the remaining two participants began active interventions at the third clinic visit (Week 5). The first clinic visit was used to assess eligibility, explain the protocol, obtain informed consent, and commence the trial. Subsequent visits were for taking measurements and intervention changeover. During these visits, blood pressure, weight, pulse rate, rescue medications, DASS-21, and PSFS data were collected. During all visits, information about the general health of participants and any adverse events was collected. At the beginning and end of the study, participants were asked to complete a Personal Wellness Index (PWI) questionnaire and blood tests. At the final clinic visit, participants were invited to provide feedback and share their experience as a study participant. Participants were also required to record daily pain scores using a VAS and any rescue medication usage. Participants were instructed to maintain their usual analgesic regimen throughout the study, whether prescribed or over the counter, taken on a standing schedule or pro re nata, with no changes to medication type or dose requested.

### Design rationale

A multiple‑baseline design (MBD) was selected for this study rather than a crossover N‑of‑1 because the combined probiotics and PEA intervention was unlikely to exhibit rapid onset/offset and any withdrawal effects were uncertain, making washout periods impractical and lengthening participant burden. In OA, symptoms fluctuate with episodic flare‑ups that may persist for weeks to months, so staggered starts across individuals support within‑person inference while helping to separate intervention onset from the natural history of flare resolution. A placebo lead‑in was used to reduce expectancy and support blinding of the transition to the active phase; two‑ and four‑week baselines were used to establish individual trajectories prior to the randomised start of active intervention (Fig. 1).

### Randomisation and blinding

Participants were randomly assigned to one of two pathways by a researcher (JB), who was removed from data collection of outcomes assessment. Randomisation was conducted in Excel using a random number generator. The schedule was provided to the industry sponsor who was providing the supplements and matched placebos. To enable double blinding, the industry sponsor labelled the bottles with a code that concealed the sequence allocation from researchers and participants. All interventions were provided in opaque capsules in tamper-evident sealed amber glass jars. In addition, the participants and outcomes assessor were also blinded to the study design.

### Treatment interventions

There were two components to the active treatment: one capsule twice daily of Metagenics Ultra Flora Intensive Care (ARTG:286746) and one capsule twice daily of Metagenics Bio Absorb PEA (ARTG:330607). Each 600 mg clear capsule of the Ultra Flora Intensive care contained *Bifidobacterium animalis* ssp. *lactis* (BB-12^®^) (5 × 10^9^CFU), *Lactobacillus rhamnosus* (LGG^®^) (10 × 10^9^CFU) and *Saccharomyces cerevisiae* (boulardii) (7.5 × 10^9^CFU). Each dark green 300 mg capsule of the Metagenics Bio Absorb PEA contained 299.9 mg of palmidrol. The dosage of 600 mg/day represents the minimum ‘loading dose’ and maximum ‘maintenance dose’, informed by the literature [29, 33]. All placebo interventions were in capsules matched for size, weight and smell, but contained only microcrystalline cellulose.

### Outcomes

#### Primary outcome

The primary outcome was pain, as measured by a 10 cm visual analogue scale VAS [34]. This was a single, unbroken 10 cm horizontal line with vertical lines at point 0 and every 1 cm thereafter. Anchors were 0 (*No pain*) to 10 (*Worst pain possible*). To facilitate intra-individual consistency, each participant was asked to nominate a personally convenient time to complete the VAS each day. Participants were issued with a diary of 14 VAS scales, two per page. Additionally, the diaries requested participants record rescue medication usage and changes in health. Participants returned the completed pain diaries at clinic visits, where a new blank diary was issued.

#### Secondary outcomes

The secondary outcomes were assessed as follows:

##### Patient specific functional scale (PSFS)

This individualised Patient Reported Outcome Measure (PROM) uses a holistic approach to function-based movement and has been shown to be valid, reliable, and responsive [35]. At the start of the trial, participants were asked to identify five daily tasks that were impacted by their chronic OA pain that they would like to monitor through the study. Each of the five tasks were assessed by the question ‘Today, how difficult do you find each of these activities?’ and scored using a 10-point scale ranging from 0 (*Do it with no problems*) to 10 (*Can’t do it at all*). The five daily tasks could also be summed to obtain a total score out of 50 and averaged to obtain an average activity score on the original 10-point scale. Data using this scale were collected at clinic visits. As participants were co-designers in this trial, their individually nominated tasks are presented in Table 1.

**Table 1:** Activities nominated by participants in the patient specific functional scales (PSFS)

Tasks	Participant 1	Participant 2	Participant 3	Participant 4
1	Getting out of bed	Sitting for too long	Sweeping the floor	Driving
2	Vacuuming	Getting up	Going up and down stairs	Throwing a ball
3	Walking	Walking	Walking	Leg raising
4	Going up and down stairs	Turning in bed	Getting out of bed	Lying on right shoulder
5	Standing more than 5 min	Lifting weights	Gardening	Work (massage therapist)

##### Personal wellness index (PWI)

The Personal Wellness Index (PWI) [36] is a 9-item self-report questionnaire that assesses subjective wellbeing across the following domains: overall life satisfaction, standard of living, health, life achievement, relationships, safety, community-connectedness, future security, and spirituality. Participants rated their satisfaction for each domain on a scale of from 0 (*No satisfaction at all*) to 10 (*Completely satisfied*). The use of the PWI has demonstrated good internal reliability with a Cronbach’s alpha of more than 0.72 in a variety of studies [37]. Scoring involves averaging across items. To standardise the scores on a scale of 100, the total score is divided by 8 (including the optional last item), then multiplied by 100. Higher scores reflect higher personal wellbeing, quality of life and mental health. Scores have been interpreted as normal (70–100), compromised (50–69), and challenged (0–49) [38].

##### Depression, anxiety and stress scale (DASS-21)

The DASS-21 [39, 40] is a 21-item instrument that measures perceptions of stress, anxiety, and depression. As a shortened version of the original DASS-42, the DASS-21 has demonstrated reliability and validity [41]. There are seven statements for each of the three domain of stress, anxiety, and depression. Statements are self-rated on a four-point Likert scale from 0 (*Did not apply to me at all*) to 3 (*Applied to me very much*,* or most of the time*). Scores were summed across items to calculate a total score for each subscale and an overall total score. Higher scores reflect higher levels of the respective constructs, while the overall total score is a measure of distress.

##### Participant study experience and success of blinding checking

At the final clinic visit, participants were asked to reflect upon their experiences in the study. The questions included whether: (a) they felt the treatment they received was of benefit, (b) they were satisfied with the way in which the study was run, (c) they felt informed about all aspects of the study, and (d) they would be willing to participate in further studies. Participants were also invited to provide in writing or verbally any comments they wished to make about the study. Participants were also asked to nominate weeks of the trial in which they believed they received the active intervention.

##### Changes in pain location and quality

Using the daily pain diaries, participants were asked to identify pain location(s) by making a mark on a diagram. Descriptions of pain quality were obtained by participants circling one or more of the following: shooting, aching, sharp, dull, burning, throbbing, radiating, gnawing, stabbing, numb, and unbearable.

##### Rescue medication usage

All participants were asked to record usage of analgesic medication in addition to the medications recorded at baseline in the daily pain diaries.

##### Changes in inflammatory markers

Participants had blood tests before and after the trial to assess safety and measure inflammatory markers. Safety measures included a full blood count that captured haemoglobin values, white blood cell values, and platelet levels. Markers of inflammation included C-Reactive Protein (CRP) [42], erythrocyte sedimentation rate (ESR) [43], and fibrinogen [44]. Markers used to assess mood included Iron [45] and vitamin D [46]. Reference ranges used were supplied by the pathology laboratory: CRP < 6.0 mg/l, ESR < 30 mm/hr, fibrinogen 1.8–8.0 g/L, serum iron 10–30umol/L and vitamin D 51–200nmol/L.

### Data analysis

Visual analysis and descriptive statistics were used to analyses the data. All data were entered into Excel spreadsheets for analysis. Excel was used to calculate minimum, maximum, mean, and standard deviation for each individual during the placebo and active intervention phases. A multiple baseline graph was generated for the daily pain VAS scores. This is a staggered time-series graph that displays data for each participant across the different phases of the study. Visual inspection of the changes in the scores when the intervention is introduced at different times was explored and reported descriptively. Secondary outcomes were analysed using time-series graphs across time points. Stata (v16) was used to generate line graphs to explore participant data across time points for the secondary outcomes. The Critical Appraisal Skills Programme Randomised Controlled Trial Standard Checklist [47] and the CONSORT 2010 checklist were used to inform the design of the study [48].

## Results

All four participants completed the study. Participants were instructed to maintain their usual analgesic regimen across study phases (prescribed or over‑the‑counter). Use of rescue medications was recorded in daily diaries and is summarised in Table 2. A participant characteristics summary is provided in Table 2.

**Table 2:** Participant characteristics

	Participant 1	Participant 2	Participant 3	Participant 4
Age	51	55	69	26
Gender	Female	Female	Female	Male
Occupation	Accounts officer	Early childhood teacher	Retired	Uni student
Locations of pain	Right knee, left knee, right wrist	Left hip, left knee, lower back, upper back	Neck, right shoulder left shoulder, right wrist and hand, left hand, upper, middle and lower back, right hip, left hip, right foot	Right ankle, right shoulder and left foot
Medication usage	As needed: Panadol Osteo (paracetamol 665 mg), Mobic (prescription medicine containing 15 mg of meloxicam)	As needed: Mersyndol (opioid prescription medicine containing 450 mg of paracetamol, 9.75 mg of codeine phosphate hemihydrate, and 5 mg of doxylamine succinate), SleepEze (1/4 tab totalling approximately 6 mg of diphenhydramine hydrochloride), Panadol Osteo (paracetamol 665 mg)	Regular prescription medicine: Escitolopram (10 mg). Following pre-trial blood test was advised to take Vitamin D	None

### Safety and adverse events

Two participants noted feelings of dry mouth despite increased water intake. For Participant 2, this symptom pre-dated the trial but was exacerbated after the first clinic visit (placebo phase) before reducing to pre-trial levels by the second clinic visit. For Participant 3, dry mouth was noted at the third clinic visit (prior to active intervention use) and gradually decreased thereafter. Both participants were invited to seek medical advice and/or leave the trial. Both declined the offer of medical advice and opted to complete the trial with the naturopath monitoring symptoms at subsequent visits.

### Primary outcome

#### VAS pain scores

**Fig. 2:**
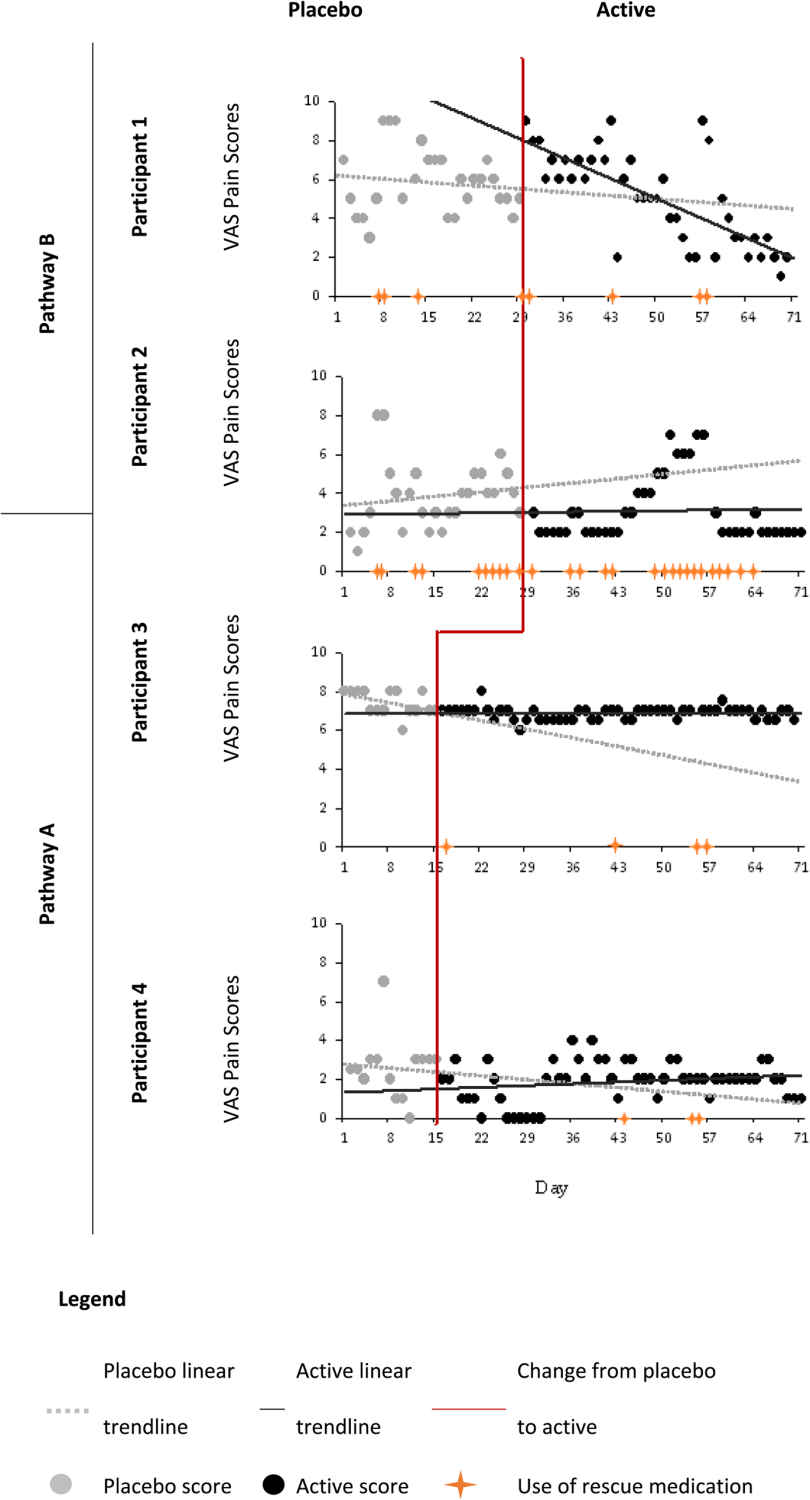
VAS pain scores

There were 280 pain score measurements taken throughout this study, which are presented in Fig. 2.

There was a downward trend for average pain scores from the placebo phase to the active phase (Table 3).

**Table 3:** Summary of VAS pain scores

	Pathway A				Pathway B			
	Participant 3		Participant 4		Participant 1		Participant 2	
	Placebo	Active	Placebo	Active	Placebo	Active	Placebo	Active
Mean	7.4	6.8	2.6	1.9	5.9	5.0	3.9	3.1
SD	0.6	0.3	1.6	1.0	1.7	2.4	1.7	1.7
Min	6	6	0	0	3	1	1	2
Max	8	8	7	4	9	9	8	7
Days in phase	15	54	14	56	27	41	27	41

#### Secondary outcome

##### Patient specific functional scale (PSFS)

**Fig. 3:**
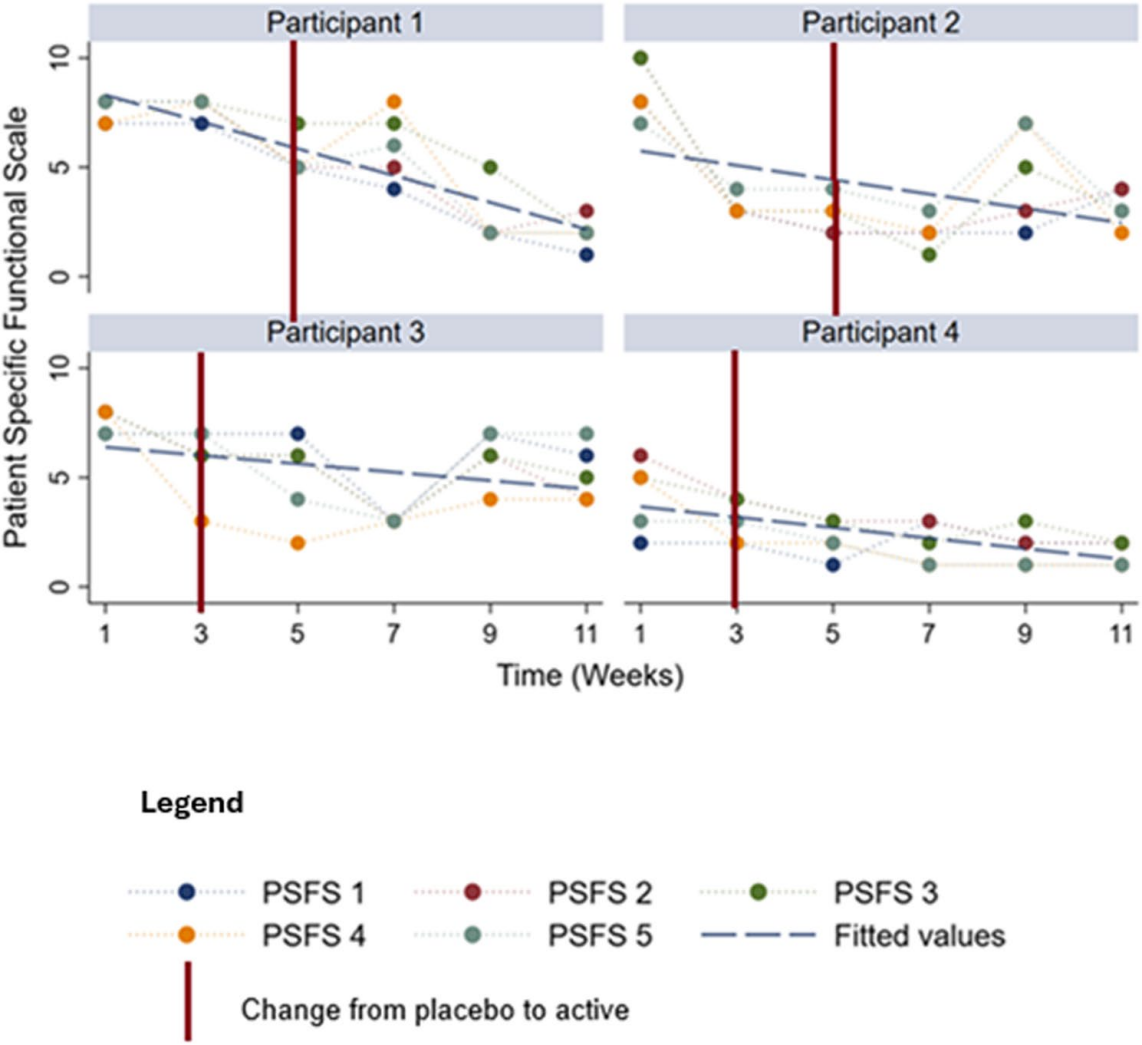
Patient-specific functional scale (PSFS) across clinic visits. Note PSFS scores for each participant across clinic visits. At visit 1, participants nominated five daily activities affected by OA pain (see Table 1). Each task is rated 0–10 (*0 = Do it with no problems*; *10 = Can’t do it at all*); lower scores indicate improved function. The plotted series show the nominated tasks and the participant’s total PSFS (sum of five tasks; 0–50) and/or mean PSFS (0–10) where indicated. The vertical red line marks the transition from placebo to active intervention; the timing differs by pathway in the multiple-baseline design

Total PSFS scores demonstrated decreased impairment and improved function for all participants (Fig. 3). Some tasks were common across multiple participants. For example, getting out of bed and stairs were common to Participant 1 and Participant 3. Walking was nominated by Participant 1, 2, and 3.

##### Personal wellbeing index (PWI)

All participants improved in PWI scores except for Participant 3 whose wellbeing score remained in the normal range at both time points (Table 4).

**Table 4:**
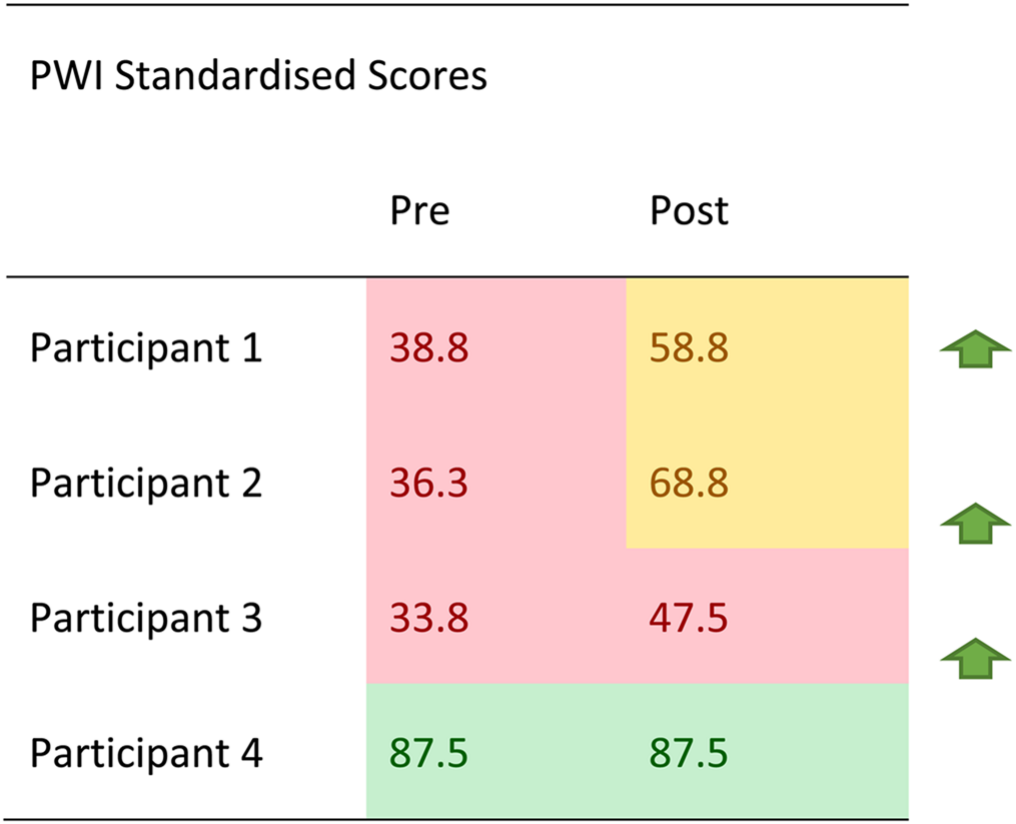
Standardised personal wellbeing index (PWI) scores pre and post intervention

Standardised scores are scaled out of 100 and interpreted as normal (70 – 100) shaded green, compromised (50 – 69) shaded amber, and challenged (0 – 49) shaded red. Green arrows indicate direction of movement of total scores across time points

Note. Standardised scores are scaled out of 100 and interpreted as normal (70–100) shaded green, compromised (50–69) shaded amber, and challenged (0–49) shaded red. Green arrows indicate direction of movement of total scores across time points.

**Fig. 4:**
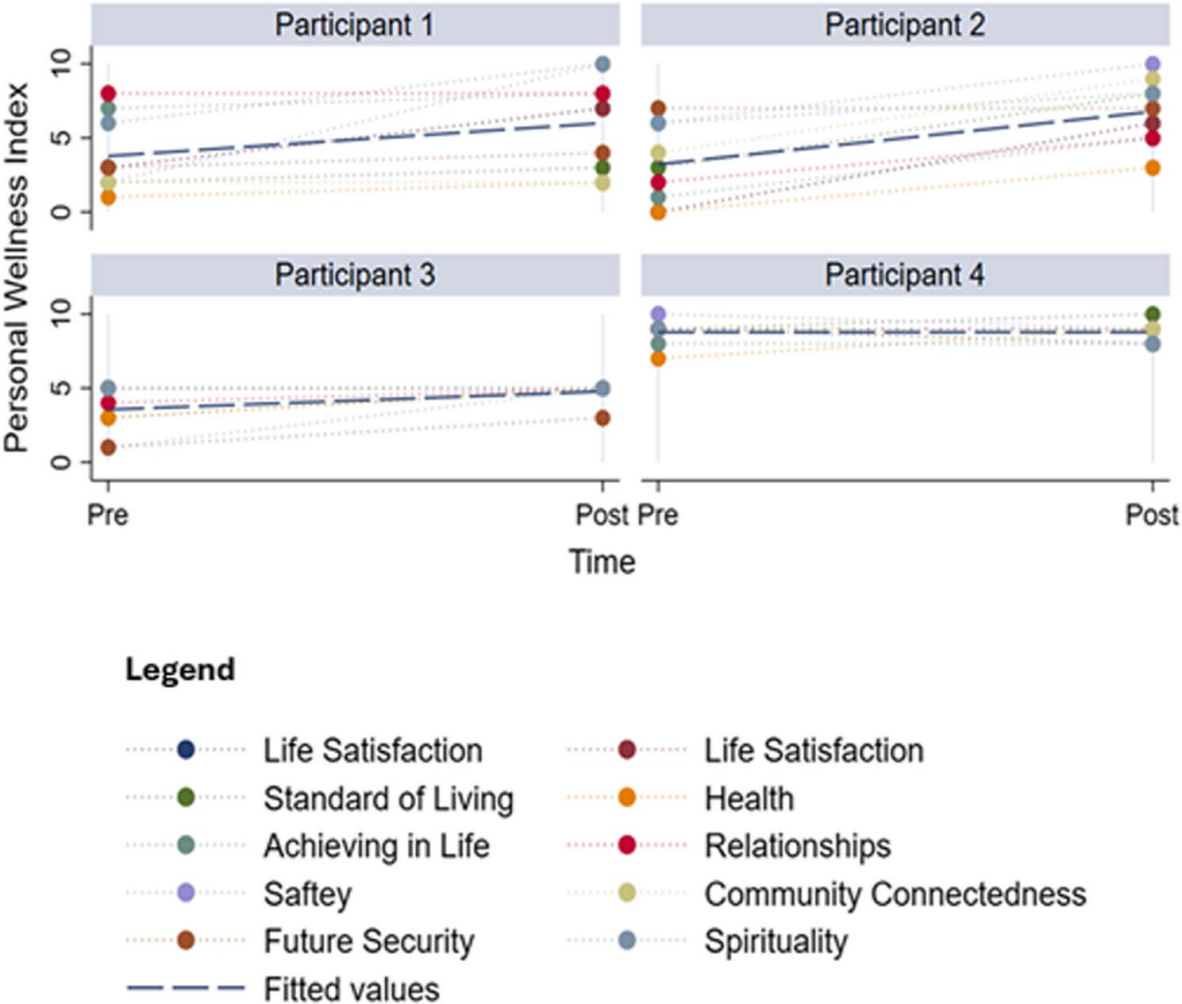
Personal wellbeing index (PWI) domain scores by participant. Note: personal wellbeing index domain scores for each participant at baseline and end of study. Domains: life satisfaction, standard of living, health, life achievement, relationships, safety, community-connectedness, future security, and spirituality. Items are rated 0–10 and displayed on a 0–100 scale; higher scores indicate better wellbeing. The horizontal line indicates the participant’s mean across domains at each time point

Figure 4 illustrates the individual domains of the PWI for each participant pre- and post-trial with a regression line fitted at the mean for each participant.

### DASS-21

**Fig. 5:**
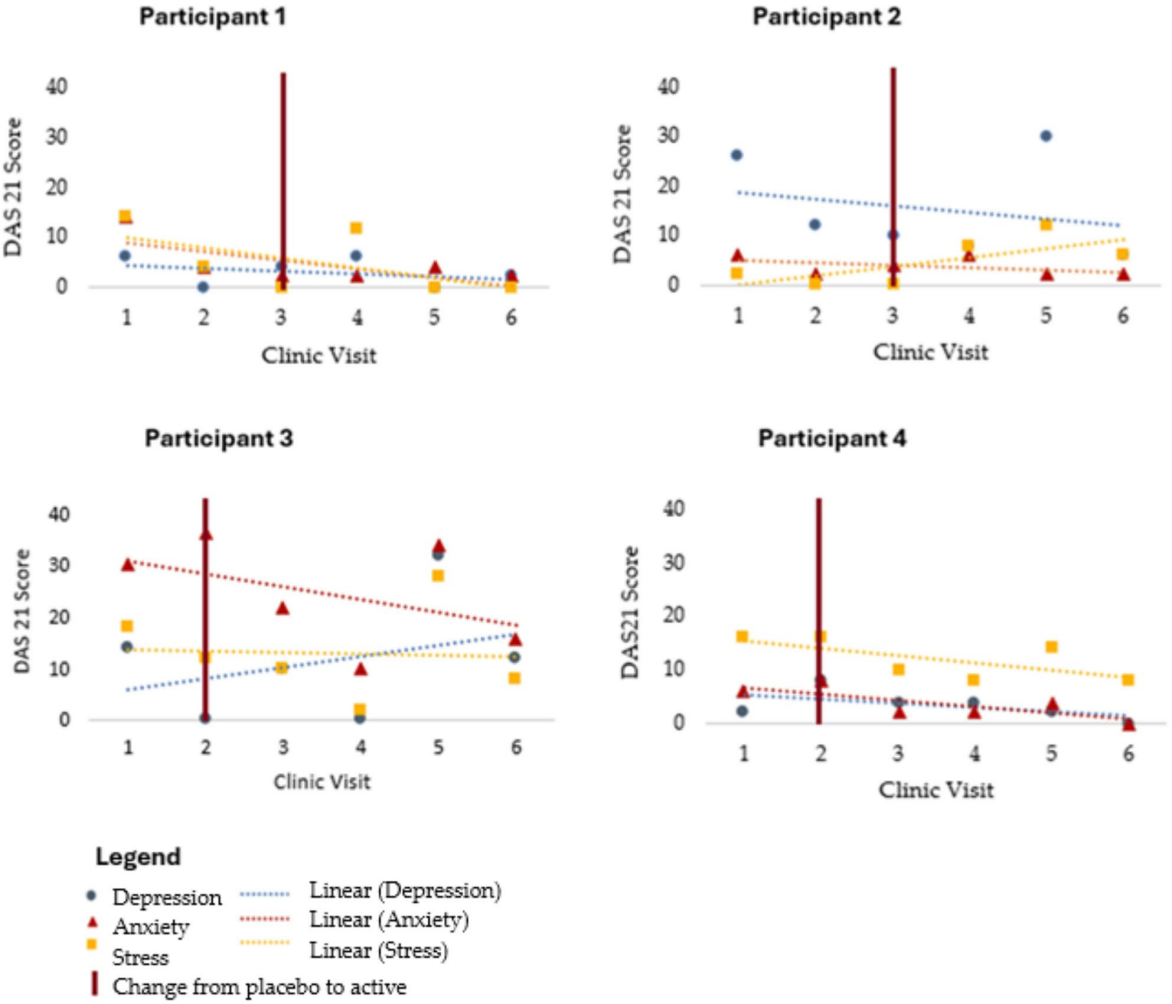
DASS-21 subscales across clinic visits. Note: depression, anxiety, and stress scale (DASS-21) subscale scores for each participant at clinic visits. Each subscale comprises 7 items scored 0–3 and summed to yield domain totals; higher scores indicate greater symptom severity. The vertical red line marks the transition from placebo to active intervention in the multiple-baseline design

Comparisons of DASS-21 scores are presented in Fig. 5. There were reductions in depression and anxiety scores for all participants but only two participants recorded reduced stress scores. Consistent with individual variability, Participant 2’s stress scores increased over the study period (Fig. 5), whereas stress declined or remained similar for the other participants. Clinic visit notes indicate that this increase in stress coincided with a period of increased personal workload.

### Participant experiences

All participants responded positively to the following questions (a) felt the treatment they received was of benefit, (b) were satisfied with the way in which the study was run, (c) felt informed about all aspects of the study, and (d) would be willing to participate in further studies. The question to assess blinding found participants were mostly inaccurate when reporting which weeks they noticed health improvements compared to when they were taking the active intervention (Table 5). Only one participant guessed they were receiving the active intervention during the placebo phase.

**Table 5:**
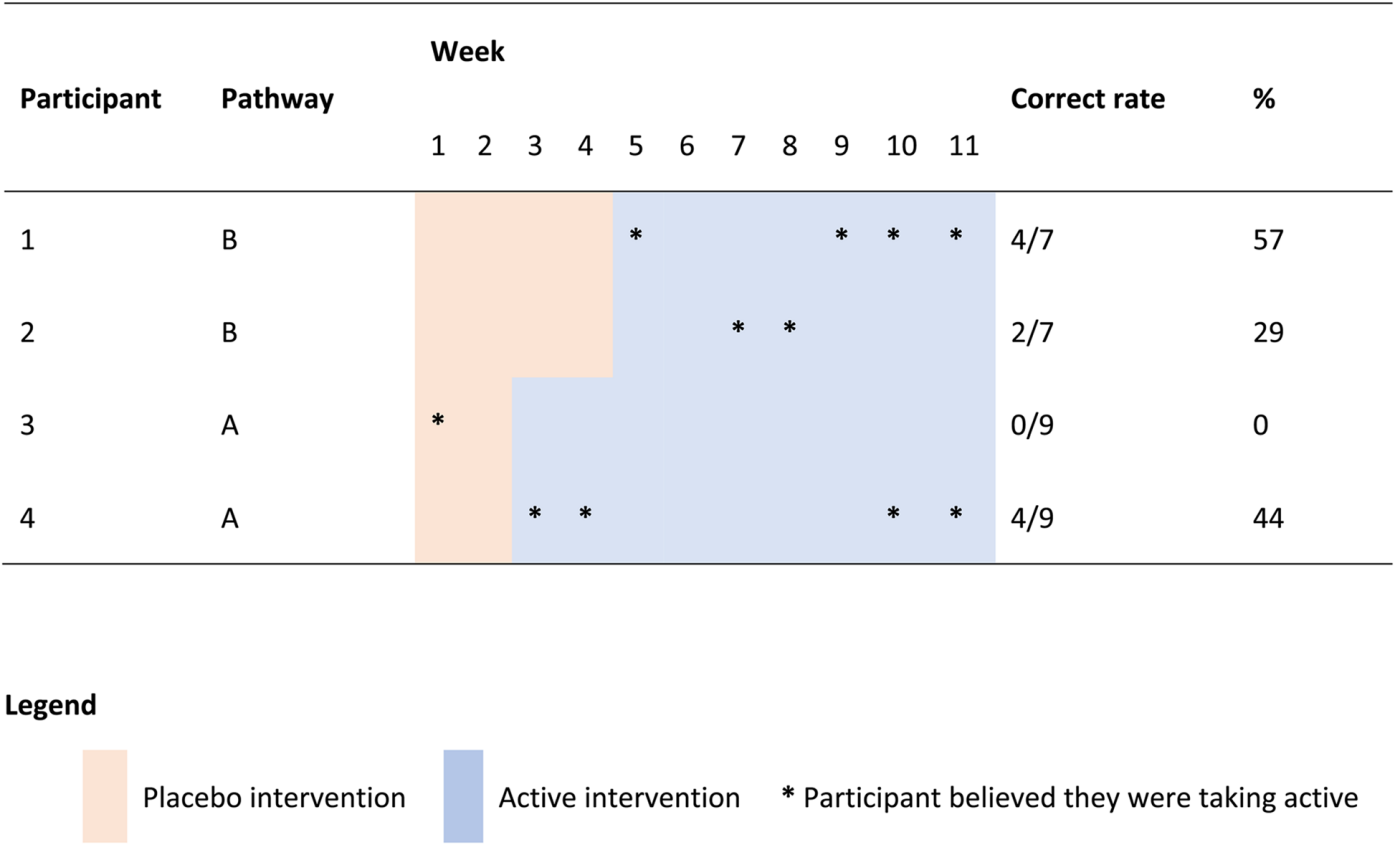
Success of blinding

### Changes in pain location and quality

Pain locations and quality varied across participants. Participant 1 recorded both wrists, both hips, and both knees as painful. Pain was described as aching, sharp, dull, burning, gnawing and on two occasions, unbearable (in the placebo intervention phase). Participant 2 identified their lower back, left hip, and left knee as painful. They described the pain as aching, dull, throbbing, radiating, gnawing, and burning. Participant 3 identified multiple sites of pain including bilateral neck, shoulders, wrists, upper/mid and lower back, bilateral hips, right knee, and right foot. They described the pain quality as shooting, sharp, aching, dull, throbbing, radiating, and numbing. Participant 4 identified their right shoulder, right foot, and occasionally left foot as being areas of concern. They mostly described the pain as aching and occasionally sharp and dull. For all patients, no changes in pain location or quality were recorded throughout the trial.

### Rescue medication usage

Participants were advised to continue rescue medication usage as needed. For Participant 1, regular pain medications prior to the trial included Panadol Osteo (2 tablets, each containing 665 mg of paracetamol) and Meloxicam (1 tablet, 15 mg of meloxicam). Participant 1 used these medications together on eight occasions across the placebo and active intervention phases.

For Participant 2, regular medications used prior to the trial included Sleep Eze, Mersyndol and Panadol Osteo. Sleep Eze is an over-the-counter medication containing 25 mg of diphenhydramine hydrochloride and is used as a sleeping aid. Mersyndol is an opioid prescription medicine containing 450 mg of paracetamol, 9.75 mg of codeine phosphate hemihydrate and 5 mg of doxylamine succinate. Participant 2 used Sleep Eze (for sleep) and Mersyndol on 25 occasions throughout the trial across the placebo and active intervention phases.

Participant 3 used Children’s Chewable Nurofen and Panadol on three occasions during the active intervention phase. Children’s Chewable Nurofen is an over-the-counter anti-inflammatory that contains 100 mg of ibuprofen per dose. The participant took two chewable tablets (totalling 200 mg of ibuprofen) for a single occasion. For the other two occasions, the participant took 2 capsules of Panadol (each capsule containing 500 mg of paracetamol) in addition to the two Children’s Chewable Nurofen.

Participant 4 used Voltaren once during the active intervention phase. Voltaren is an over-the-counter anti-inflammatory that contains 25 mg of diclofenac potassium. The dosage taken was 2 capsules (totalling 50 mg of diclofenac potassium). Participant 4 experienced cold and flu symptoms during the active intervention phase and took unspecified over-the-counter medication for two consecutive days to alleviate symptoms of congestion.

### Changes in inflammatory and mood markers

Blood test results are presented in Table 6. The fibrinogen levels for Participant 2 were mildly elevated but did not require intervention according to the participant’s doctor. All participants recorded an increase of vitamin D. Participant 3 was initially deficient in vitamin D (33nmol/L) and following advice from their doctor was supplemented with 20mcg of calcifediol monohydrate daily resulting in 157nmol/L post-trial. All participants recorded decreased iron levels post-trial. Participant 1 recorded increases in CRP, ESR, and fibrinogen levels post-trial. For Participant 2 CRP levels remained the same while ESR levels increased, and fibrinogen decreased. Participant 3 recorded consistent CRP and fibrinogen levels while ESR levels decreased. Participant 4 recorded consistent results for CRP and ESR and a marginal fibrinogen decrease.

**Table 6:** Blood test results

Participant	CRP (< 6 mg/L)		ESR (< 30 mm/hr)		Fibrinogen (1.8–4.0 g/L)		Serum Iron (10–30 umol/L)		Vit D (51–200 nmol/L)	
	Pre	Post	Pre	Post	Pre	Post	Pre	Post	Pre	Post
1	4	5.1	8	10	3.7	3.9	13	8	58	66
2	4	4	16	18	4.4	3.9	13	10	61	82
3	4	4	13	8	3.7	3.7	23	7	39	157
4	4	4	2	2	2.7	2.6	22	20	68	76

## Discussion

While the primary outcome of reduced pain was clearly demonstrated in only one participant, the combination of probiotics and PEA appears to be associated with improved mobility for all participants according to their chosen personalised outcome measures. Furthermore, the combination of probiotics and PEA were associated with increases in wellbeing for three of the four participants. These variabilities underscore the importance of personalised approaches to pain management, particularly in a heterogeneous condition like chronic pain in individuals with OA. By focusing on individual participants and empowering them to select their own functional patient-reported outcomes, this study facilitated careful observation of individual changes over an 11 week period, thereby providing insights into the nuances of how the interventions could affect different people in unique ways [49].

The 11-week observation window should be interpreted in the context of OA’s episodic course. Flare-up periods can arise unpredictably and may persist for weeks to months before resolving, which means symptom trajectories observed within our timeframe could overlap with the natural rise and fall of disease activity [50]. In our design, baselines were 2 or 4 weeks and active phases were ~ 7–9 weeks depending on pathway, which strengthens within‑participant inference but cannot fully exclude overlap between intervention onset and spontaneous flare resolution. These considerations are consistent with our cautious interpretation of the pain and PROM findings and support the need for longer follow-up and/or repeated AB sequences in future studies. Interpreting the present findings therefore requires caution. The primary outcome showed a clear VAS reduction in only one participant, with modest or variable changes in others; functional PROMs and wellbeing improved for several but not all participants; and CRP/ESR were largely unchanged. Given the descriptive analytic approach, small n, and individual variability (including rescue‑medication use across phases), the positive signals observed here should be viewed as preliminary and hypothesis‑generating rather than confirmatory.

The use of PROMs offered a comprehensive evaluation of intervention from the perspective of the individual participant, capturing insights that would have been omitted by purely objective measures that focus on comparison with population means. Inclusion of the PROMs reflects the growing understanding of how personalised, multimodal approaches can influence wellbeing in individuals with chronic pain [51, 52]. Improvements in function and mood are consistent with research suggesting chronic pain and psychological distress are linked with both affecting individual wellbeing [6]. A pilot study that investigated chronic pain rehabilitation similarly reported that rehabilitation perceived as personally meaningful was significantly associated with improved wellbeing, but not directly with reduced pain [52]. This may suggest that integrating personal values and addressing markers of wellbeing into treatment plans as outcomes may enhance quality of life which may be particularly beneficial for individuals with chronic pain. For example, in this study, participants independently nominated common tasks (e.g. getting out of bed, walking, and climbing stairs) which demonstrated a trend towards improvement. The successful completion of such daily tasks has been associated with greater life satisfaction and wellbeing [53].

The objective outcome measure of blood testing found decreased iron levels and increased vitamin D for all participants. While Participant 3 was supplemented with vitamin D, these results raised questions about the impact of probiotic and PEA supplementation on iron and vitamin D absorption. Current research suggests that probiotics can increase serum levels of vitamin D which can then decrease inflammation [54, 55]. One study suggested *Lactobacillus plantarum 299v* was effective for improving iron status and vigor as it facilitated iron absorption [56]. While the impact of PEA on the gastrointestinal system is emerging, there are no studies yet assessing its impact on vitamin D or iron levels.

The subjective improvements in function were not correlated with decreased inflammatory markers of CRP and ESR. With the exception of Participant 3 who recorded a decrease in ESR, all participants recorded stagnant or increased levels of CRP and ESR. This may suggest that the intervention’s potential benefits are not mediated by these markers and may benefit from further investigation into other mechanisms of action. Several factors may explain why CRP and ESR did not decrease in parallel with functional gains. First, both CRP and ESR are non-specific, systemic markers and may be relatively insensitive to the predominantly local low‑grade synovitis typical of OA and modest intra‑articular inflammatory changes may not translate into systemic shifts at the time of sampling. Second, the 11‑week observation window and timing of blood draws may have missed transient changes or overlapped with unrelated concurrent influences on these markers. Third, individual variability and concomitant behaviours/medications can affect CRP/ESR independently of joint symptoms. Together, these considerations suggest that additional and/or more sensitive biomarkers (e.g., cytokines) and/or synovial or imaging endpoints may be needed in future work to capture mechanistic change.

To contextualise the magnitude of pain change, anchor‑based thresholds for a minimally important improvement on a 10 cm VAS in rheumatology/OA cohorts are commonly in the range of about 1–2 cm (e.g., ≈ 1.37 cm reported in adult rheumatology populations) [57], recognising that exact values vary by method and anchor. The Initiative on Methods, Measurement, and Pain Assessment in Clinical Trials (IMMPACT) consensus suggests that ≥ 30% reduction reflects a moderately important improvement and ≥ 50% a substantial improvement in chronic pain trials [58]. Future studies of this intervention should pre‑specify both absolute (cm) and relative (%) responder definitions (e.g., OMERACT‑OARSI style criteria [50]) and power analyses accordingly.

Rescue-medication use, recorded in daily diaries and summarised in Sect. 3.3.6, varied by participant and occurred in both placebo and active phases, which may have attenuated or accentuated within-phase VAS differences in some cases. We therefore interpret the modest mean VAS changes with caution, given rescue-medication usage across phases and the multiple-baseline design’s limited ability to exclude natural symptom fluctuation (e.g., Participant 1).

The role of the microbiome in OA and its potential modulation by probiotics is a burgeoning area of research. The gut microbiome has been implicated in systemic inflammation, immune modulation, and mood regulation, all factors that are relevant to OA pathology [59]. The link between microbial diversity and the endocannabinoid system is gaining attention [60–65], however there is limited research investigating the link between PEA and its impact on the human microbiome [66]. It has been proposed that PEA may attenuate gut microbial diversity through gastrointestinal permeability, mRNA expression, lipopolysaccharide activation, and lipogenesis [65, 67, 68]. In humans, PEA was found to affect reward behaviours, anhedonia, amotivation, and influence gut microbial diversity via its influence in the endocannabinoid system [60]. While PEA may indirectly modulate the endocannabinoid system, it is not a classical endocannabinoid, and cannabinoid pharmacotherapies were beyond the scope of this study.

Future studies could consider microbiome analyses to further explore the role of the individual gut microbiome and the link between microbial diversity and the endocannabinoid system to determine whether specific microbial profiles are predictive of individual treatment response. Additionally, the inclusion of biomarkers including IL-6 may provide a more comprehensive understanding of the inflammatory pathways involved in OA. Finally, further research into the impact of PEA on nutritional status may be warranted.

## Limitations

There are several limitations associated with this study. Firstly, the use of a MBD study is recognised as a pragmatic alternative to RCTs and arguably more clinically effective for smaller population samples [69]. However, the small sample size indicates that findings may also be preliminary and hypothesis-generating. Internal validity may benefit from ensuring baseline data is stable before the implementation of the intervention, which can be captured with a MBD study [69]. Conversely, however, such designs have limited representativeness as they are designed to look at effects in individuals, rather than means in populations. Consequently, the inferences are limited to the individuals under investigation and should not be used to infer to populations, without further testing. The use of PROMs is designed to capture individual experiences but may be influenced by individual life events that may impact outcomes. For example, one of the study participants (Participant 4) had their normal daily routine altered due to work placements which began the week before the trial started and continued post-trial conclusion which may have influenced their normal daily pain scores. Despite the positive findings presented in this study, it is crucial to interpret these findings with caution as observed improvements may also represent natural fluctuations of OA symptoms or the resolution of previous flare-ups which may occur over a period of weeks [49]. Additionally, although the staggered multiple-baseline design provides some protection against temporal confounding, we did not prospectively capture flare events with a dedicated instrument, and baseline stability was constrained by pragmatic scheduling (two or four weeks). Longer observation periods, explicit flare diaries or instruments, and extended or repeated AB sequences would allow better separation of intervention effects from the natural history of flare onset and resolution. Finally, our sample size (*n* = 4) limits inference to individuals rather than populations; larger studies are required to estimate population-level effects and assess heterogeneity of response.

Negative and null findings were also observed. The primary outcome (daily pain) showed a clear reduction in only one participant, with more modest or variable patterns in others. For mood, stress scores increased for Participant 2 during the study (see Fig. 5), whereas depression and anxiety generally declined. Objective inflammatory markers (CRP/ESR) were stagnant or increased for most participants, and one participant’s wellbeing (PWI) did not improve across the trial window. These mixed results underscore heterogeneity of response and the need for cautious interpretation.

The potential for a significant placebo effect must be acknowledged. Although the calculated blinding success rate was moderate, only one participant correctly guessed they were receiving the active intervention during the placebo phase. Furthermore, expectancy effects and the regularly scheduled personalised attention and monitoring embedded in the study design may have contributed to perceived or actual improvements, independent of the intervention. While compliance of interventions was able to be monitored through the use of counting remaining capsules at subsequent visits, there was no procedure in place to ensure participants recorded VAS scores as directed which could influence data analysis.

More generally, conclusions from this multiple-baseline, *n* = 4 design are constrained by the descriptive analytic approach, short observation window, and potential expectancy/Hawthorne effects. Inferences are appropriately limited to the individual participants studied and should not be extrapolated to populations without further testing in larger samples and with longer follow-up. Finally, the absence of a long-term follow-up limits our understanding of the sustained effects of the intervention for these individuals.

## Conclusions

This multiple‑baseline study highlights the feasibility of individualised assessment in OA and suggests that a probiotics‑plus‑PEA regimen may support function and wellbeing for some individuals. However, effects on pain and systemic inflammatory markers were modest or inconsistent, and inferences are limited by the small sample, short observation window, and descriptive analyses. Future trials should incorporate longer follow‑up windows and prospective flare capture, given the potential for multi‑month OA flares to overlap with treatment effects. Larger, controlled studies with pre‑specified clinically meaningful responder thresholds and mechanistic endpoints are needed to determine population‑level efficacy and clarify modes of action.

## Data Availability

The datasets used and/or analysed during the current study are available from the corresponding author on reasonable request.
